# Glioma-Associated Stromal Cells Stimulate Glioma Malignancy by Regulating the Tumor Immune Microenvironment

**DOI:** 10.3389/fonc.2021.672928

**Published:** 2021-04-29

**Authors:** Xiangming Cai, Feng Yuan, Junhao Zhu, Jin Yang, Chao Tang, Zixiang Cong, Chiyuan Ma

**Affiliations:** ^1^ School of Medicine, Southeast University, Nanjing, China; ^2^ School of Medicine, Nanjing University, Nanjing, China; ^3^ Department of Neurosurgery, Jinling Hospital, Nanjing, China; ^4^ School of Medicine, Nanjing Medical University, Nanjing, China

**Keywords:** glioma, glioma-associated stromal cell, immune check points, M2 macrophages, tumor microenvironment

## Abstract

**Background:**

The glioma-associated stromal cell (GASC) is a recently identified type of cell in the glioma microenvironment and may be a prognostic marker for glioma. However, the potential mechanisms of GASCs in the glioma microenvironment remain largely unknown. In this work, we aimed to explore the mechanisms of GASCs in gliomas, particularly in high-grade gliomas (HGG).

**Methods:**

We used glioma datasets from The Cancer Genome Atlas (TCGA) and the Chinese Glioma Genome Atlas (CGGA). We utilized the Single-sample Gene Set Enrichment Analysis (ssGSEA) algorithm to discriminate between patients with high or low GASC composition. The xCELL and CIBERSORT algorithms were used to analyze the composition of stromal cells and immune cells. Risk score and a nomogram model were constructed for prognostic prediction of glioma.

**Results:**

We observed for the first time that the levels of M2 macrophages and immune checkpoints (PD-1, PD-L1, PD-L2, TIM3, Galectin-9, CTLA-4, CD80, CD86, CD155, and CIITA) were significantly higher in the high GASC group and showed positive correlation with the GASC score in all glioma population and the HGG population. Copy number variations of DR3 and CIITA were higher in the high-GASC group. THY1, one of the GASC markers, exhibited lower methylation in the high GASC group. The constructed risk score was an independent predictor of glioma prognostics. Finally, a credible nomogram based on the risk score was established.

**Conclusions:**

GASCs stimulate glioma malignancy through the M2 macrophage, and are associated with the level of immune checkpoints in the glioma microenvironment. The methylation of THY1 could be used as prognostic indicator and treatment target for glioma. However, further studies are required to verify these findings.

## Introduction

Glioma is the most common primary malignant tumor of the central nervous system, and it generally has a poor prognosis. The World Health Organization (WHO) classified gliomas into grades I-IV, with grades III and IV indicating high-grade gliomas (HGG) (1). The current treatments for HGG involve tumor resection, radiotherapy (RT), and temozolomide (TMZ), but this strategy has not yielded optimal effects (2).

Immunotherapy has been extensively studied for human malignant tumors in the past few years (3). However, due to the “immune-cold” phenotype and inner complexity of glioma (4), only a minority of glioma patients benefit from immune checkpoint (ICP) inhibitors (5). Researchers are deepening our understanding of the complex interactions between glioma and the immune system and trying to maximize the effectiveness of immunotherapy for glioma (6).

The glioma-associated stromal cell (GASC) is a recently identified important stromal cell in the glioma microenvironment, with potential value for prognostic prediction and therapeutic perspectives (7). The available evidence indicates that GASCs facilitate angiogenesis, invasion, and tumor growth (7). However, the potential mechanisms of GASCs remain largely unknown.

**Figure 1:**
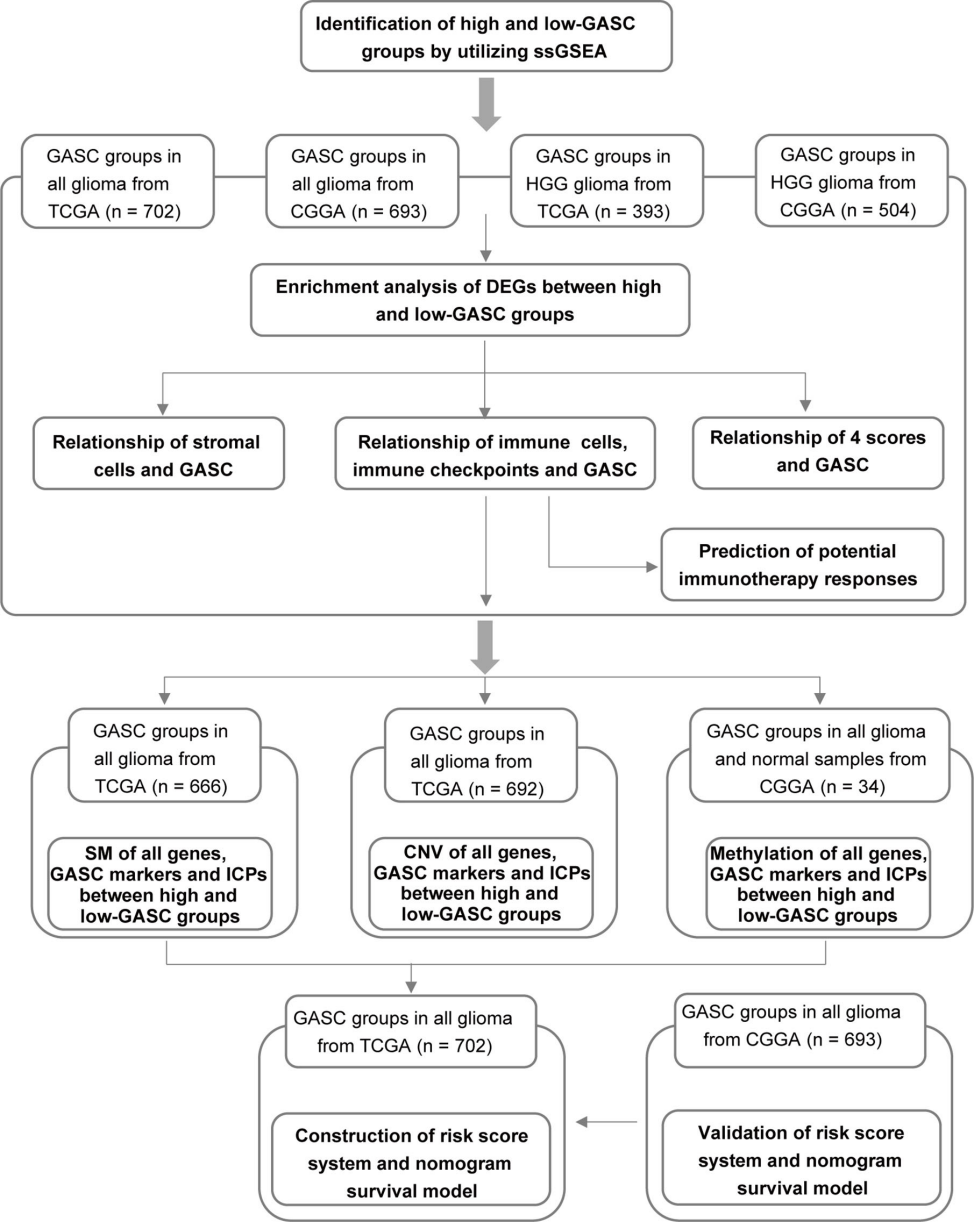
Flow diagram of this investigation. HGG, high-grade glioma; DEGs, differentially expressed genes; 4 scores, stemness, mesenchymal-EMT, tumorigenic cytokine, and angiogenic activity scores; SM, somatic mutations; CNV, copy number variations; ICPs, immune checkpoints.

We aimed to identify the underlying mechanisms of GASCs in a glioma microenvironment, particularly in HGG. **Figure 1** illustrates the workflow of the study.

## Materials and Methods

### Glioma Datasets

The Cancer Genome Atlas (TCGA, https://portal.gdc.cancer.gov/) and Chinese Glioma Genome Atlas (CGGA, www.cgga.org.cn/) are public databases. The mRNA sequencing data and clinical information data for 702 glioma samples from TCGA and 693 glioma samples from CGGA were downloaded. Among these samples, 393 samples from TCGA and 504 samples from CGGA were high-grade glioma (HGG). The somatic mutation data for 666 glioma samples from TCGA were downloaded. The copy number variation data for 692 samples from TCGA were downloaded from the UCSC Xena Project database (http://xena.ucsc.edu/). For methylation analysis, the methylation data for 34 samples from CGGA and mRNA sequencing data for 325 samples were downloaded from CGGA.

### ssGSEA Analysis

A gene set of GASC markers (**Table S1**) was obtained from Clavreul **et al. (7). Enrichment scores for GASCs were separately calculated for each sample with the Single-sample Gene Set Enrichment Analysis (ssGSEA) algorithm. We also used the ssGSEA algorithm to calculate the stemness score (8), mesenchymal-epithelial-to-mesenchymal transition (EMT) score (9), tumorigenic cytokine score (10) and angiogenic activity score (11) based on the corresponding gene sets (**Table S1**). The “GSVA” R package (version 1.34.0) was applied to conduct an ssGSEA analysis.

### Principle Component Analysis (PCA)

PCA was used to show the differentiation of high- and low-GASC groups and was visualized with the “ggfortify” R package (version 0.4.11).

### Differential Analysis of Expressed Gene

We used Morpheus (https://software.broadinstitute.org/morpheus) to identify significantly differentially expressed genes (DEGs) between the high- and low-GASC groups. *P* < 0.05 and |log2 FC (fold-change)| ≥ 1 were selected as the cutoff values for statistically significant DEGs. A heatmap of DEG expression was produced by the “pheatmap” R package (version 1.0.12).

### Functional Annotation

To reveal the probable biofunctions and signaling pathways that were correlated with the DEGs, we performed Gene Ontology (GO) annotations enrichment analysis, Kyoto Encyclopedia of Genes and Genomes (KEGG) pathway analysis and enrichment analysis, and Gene Set Enrichment Analysis (GSEA) using the “clusterProfiler” (12) package (version 3.14.3) in R. Adjusted *p* < 0.05 was selected as the cutoff criterion.

### xCELL Analysis and Cell Type Identification by Estimating Relative Subsets of RNA Transcripts (CIBERSORT) Analysis

xCell is an R package (version 1.1.0) that estimates the comprehensive levels of 64 cell types, which include 14 stromal cells. CIBERSORT can accurately quantify the abundance scores of 22 types of immune cells for each sample. We applied xCELL and CIBERSORT to separately calculate the abundance scores for stromal cells and immune cells in glioma samples.

### Analysis of Somatic Mutations and Copy Number Variations

The somatic mutations of glioma samples from TCGA were calculated and visualized by the “Maftools” R package (version 2.2.10) (13). The copy number variations were visualized by the “ComplexHeatmap” R package (version 2.2.0).

### Prediction of the Immunotherapy Response

The Tumor Immune Dysfunction and Exclusion (TIDE) algorithm (14) was employed to predict the clinical response of immune checkpoint inhibitors for each glioma sample.

### Construction of Prognostic Model

The glioma datasets from TCGA and CGGA were used separately as a training dataset and validation dataset during the construction of the prognostic model. In the filtering process, least absolute shrinkage and selection operator (LASSO) regression analysis was applied to filter input parameters with *p* < 0.05. The input parameters included GASC score, GASC markers, immune checkpoints, stemness score, mesenchymal-EMT score, tumorigenic cytokine score, angiogenic activity score, stromal cell scores, and immune cell scores. Then, multivariate Cox regression analysis was conducted, and the risk score for glioma was computed *via* this formula: risk = score $\sum_{i=1}^{n}\beta_{i}\times X_{i}.X_{i}$ indicates the input parameter of multivariate regression analysis, and *β_i_* represents the coefficient of *X_i_*. Risk score and clinicopathological features were used to construct a prognostic model with uni- and multivariate Cox regression analysis. A nomogram was built to show the prognostic model. Receiver operating characteristic (ROC) curve analysis was conducted to evaluate the effect of the prognostic model in the training and validation datasets. “glmnet” (version 4.1), “rms” (version 6.1.0), and “timeROC” (version 0.4) R packages were used for the construction of prognostic model.

### Statistical Analysis

All statistical analyses were performed utilizing R software (version 3.5.1), and statistical significance was set at *p* < 0.05. Comparisons between 2 continuous variables were evaluated by Student’s *t*-test and one-way ANOVA with ≥ 3 variables. Boxplots and bar charts were utilized to display these comparisons using the “ggplot” R package (version 3.3.3). The chi-square test or Fisher’s exact test were used for comparisons of categorized variables. The Kaplan-Meier approach was conducted for survival analysis, and the log-rank test was used to compare the overall survival (OS). Spearman correlation analysis was applied to evaluate two continuous variables, and the data were visualized with “ggplot” and “corrgram” (version 1.13) R packages. Univariate Cox regression was applied to identify potential predictors of survival, and the data were displayed with “forestplot” (version 1.10.1) R package.

## Results

### Identification of High- and Low-GASC Groups With ssGSEA

**Figure 2:**
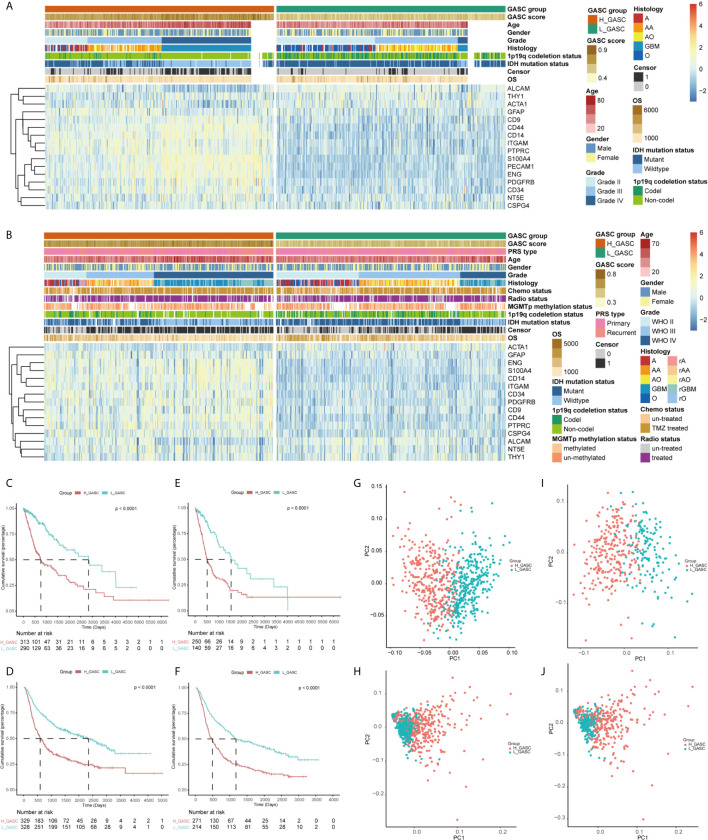
Identification of high- and low-GASC groups. **(A, B)** Heatmap of GASC markers in all glioma population (**A** for TCGA and **B** for CGGA). **(C–F)** Kaplan-Meier overall survival (OS) curves for samples in high- and low-GASC groups from all glioma population (**C** for TCGA and **D** for CGGA) and high-grade glioma population (**E** for TCGA and **F** for CGGA). **(G–J)** Principal component analysis of high- and low-GASC groups from all glioma population (**G** for TCGA and **H** for CGGA) and high-grade glioma population (**I** for TCGA and **J** for CGGA).

To analyze the potential mechanisms of GASCs in the glioma microenvironment, we obtained mRNA sequencing data for 702 samples from TCGA and 693 samples from CGGA, and then calculated the GASC score for each sample using the ssGSEA algorithm (**Figure 1**). Samples from TCGA and CGGA were classified separately into high- or low-GASC groups according to the median of the GASC score. Information for the high- and low-GASC groups is shown in **Figures 2A, B** and **Table 1**. Separate classification was also performed for 393 HGG samples from TCGA and 504 HGG from CGGA into high- and low-GASC groups using the same method. K-M curves were drawn, and the results revealed that a higher GASC score was associated with worse OS in all glioma population and the HGG population (*p* < 0.0001; **Figures 2C–F**). PCA showed robust differences in the expression portraits of the GASC markers between the high- and low-GASC groups (**Figures 2G–J**).

**Table 1:** Correlations between GASC groups and clinical characteristics in glioma patients.

Characteristic	TCGA			CGGA		
	H_GASC	L_GASC	*p*-value	H_GASC	L_GASC	*p*-value
All cases	351	351		346	347	
Age (yeas)	51.11 ± 15.89	43.25 ± 13.54	<0.001*	45.06 ± 13.30	41.51 ± 11.15	0.003*
Gender			0.126			0.792
Female	123	132		149	146	
Male	193	161		197	201	
Grade			<0.001*			<0.001*
Grade II	65	151		64	124	
Grade III	114	127		102	153	
Grade IV	137	15		180	69	
Histology			<0.001*			<0.001*
A + rA	28	27		46	73	
AA + rAA	69	45		70	82	
AO + rAO	21	66		25	57	
GBM + rGBM	137	15		180	69	
O + rO	20	97		17	43	
PRS type						0.006*
Primary				193	229	
Recurrent				153	118	
1p19q codeletion status			<0.001*			<0.001*
Codel	28	141		44	101	
Non-codel	301	194		267	211	
IDH mutation status			<0.001*			<0.001*
Mutant	139	289		134	222	
Wildtype	187	47		190	96	
MGMTp methylation status						0.696
methylated				158	157	
un-methylated				110	117	
Radiotherapy status						0.219
treated				259	251	
un-treated				61	75	
Chemotherapy status						0.013
TMZ treated				257	229	
un-treated				67	94	

A, astrocytoma; O, oligodendroglioma; AA, anaplastic astrocytoma; AO, anaplastic oligodendroglioma; GBM, glioblastoma; r, recurrence; PRS type, primary-recurrent-secondary type; TMZ, temozolomide; *p < 0.05.

### Enrichment Analysis of DEGs Between the High- and Low-GASC Groups

**Figure 3:**
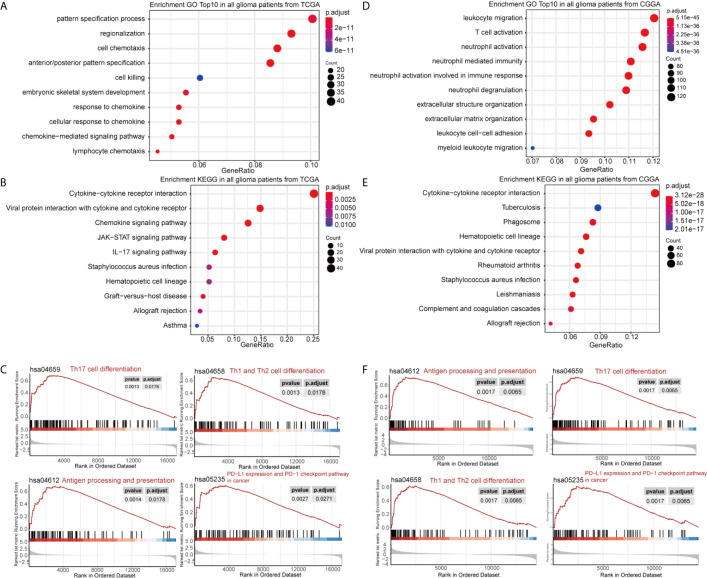
Functional annotation of upregulated DEGs between the high- and low-GASC groups from all glioma population. **(A, B)** GO analysis **(A)** and KEGG pathway analysis **(B)** of up-regulated DEGs from TCGA data. **(C)** GSEA analysis of genes from TCGA data. **(D, E)** GO analysis **(D)** and KEGG pathway analysis **(E)** of up-regulated DEGs from CGGA data. **(F)** GSEA analysis of genes from CGGA data.

**Figure 4:**
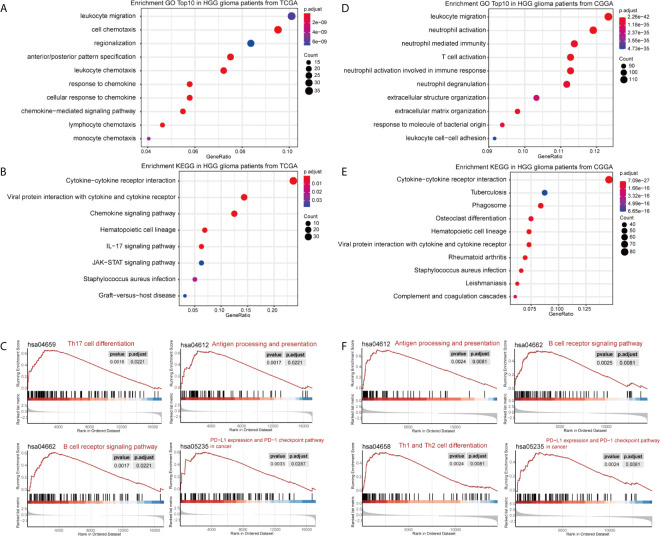
Functional annotation of upregulated DEGs between the high- and low-GASC groups from the high-grade glioma population. **(A, B)** GO analysis **(A)** and KEGG pathway analysis **(B)** of up-regulated DEGs from TCGA data. **(C)** GSEA analysis of genes from TCGA data. **(D, E)** GO analysis **(D)** and KEGG pathway analysis **(E)** of up-regulated DEGs from CGGA data. **(F)** GSEA analysis of genes from CGGA data.

DEGs between the high- and low-GASC groups were identified with the Morpheus webtool (**Figure S1**). Functional enrichment revealed a significant association between DEGs and immune-related terms. Biological process (BP) terms enriched in the GO analysis included “lymphocyte chemotaxis” and “neutrophil activation” in all glioma population (**Figures 3A, D** and **Table S2**) and the HGG population (**Figures 4A, D** and **Table S2**). The “JAK-STAT signaling pathway” and “IL-17 signaling pathway” were enriched in the KEGG analysis in all glioma population (**Figures 3B, E** and **Table S3**) and the HGG population (**Figures 4B, E** and **Table S3**). GSEA analysis revealed immune-related terms such as “Antigen processing and presentation” and “PD-L1 expression and PD-1 checkpoint pathway” in all glioma population (**Figures 3C, F** and **Table S6**) and the HGG population (**Figures 4C, F** and **Table S6**).

### Correlation of Stemness, Mesenchymal-EMT, Tumorigenic Cytokine, and Angiogenic Activity Scores With GASCs

**Figure 5:**
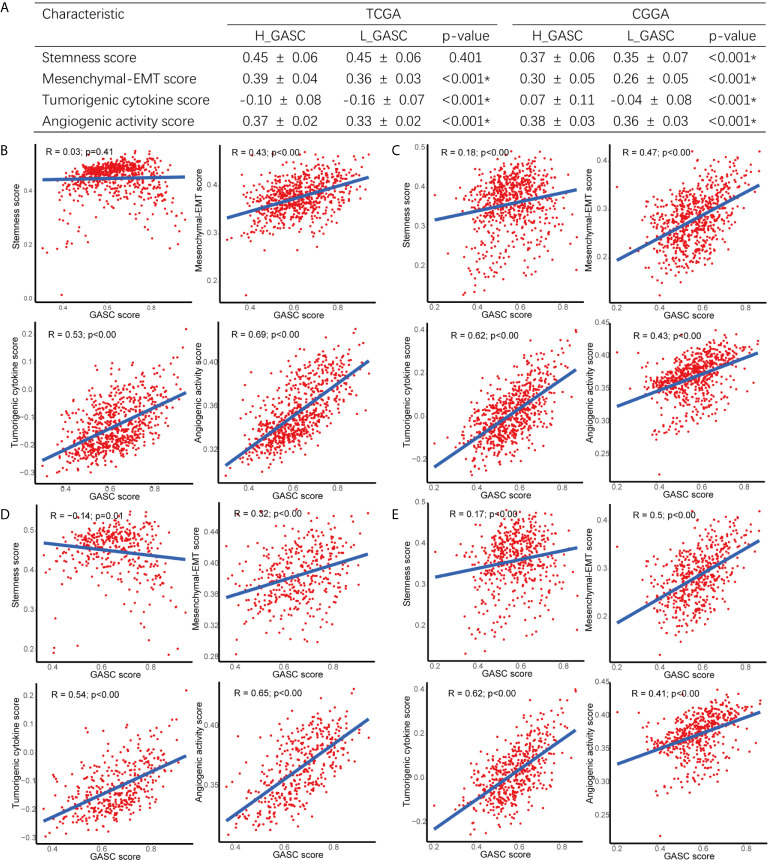
Correlations between GASCs and Stemness, Mesenchymal-EMT, Tumorigenic cytokine, and Angiogenic activity scores. **(A)** Correlations between GASC groups and stemness, mesenchymal-EMT, tumorigenic cytokine and angiogenic activity scores. **(B–E)** Scatterplot of GASC score and 4 scores in all glioma population (**B** for TCGA and **C** for CGGA) and high-grade glioma population (**D** for TCGA and **E** for CGGA). *Statistical significance.

To explore the potential mechanisms of GASCs in glioma, we also calculated stemness, mesenchymal-EMT, tumorigenic cytokine, and angiogenic activity scores for each glioma sample using the ssGSEA algorithm (**Table S1**). The results showed that mesenchymal-EMT, tumorigenic cytokine, and angiogenic activity scores were significantly higher in the high-GASC group (**Figure 5A**), and were positively correlated with the GASC score in all glioma population (**Figures 5B, C**) and the HGG population (**Figures 5D, E**).

### Associations Between GASCs and Stromal Cells

**Figure 6:**
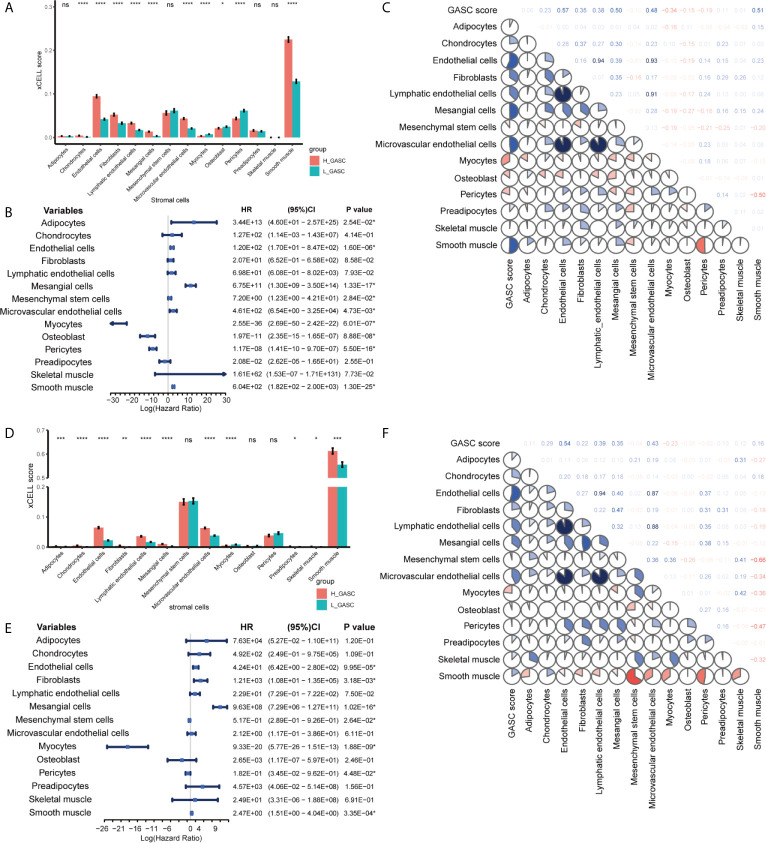
Associations between GASCs and stromal cells in all glioma population. **(A, D)** Bar charts illustrating the differences of xCELL scores between high- and low-GASC groups (**A** for TCGA and **D** for CGGA). ns: *p* > 0.05, *p ≤ 0.05, **p ≤ 0.01, ***p ≤ 0.001, ****p ≤ 0.0001. **(B, E)** Forest plots of univariate Cox regression analysis of stromal cells (**B** for TCGA and **E** for CGGA). **(C, F)** Correlograms of GASC score and stromal cells intercorrelation (**C** for TCGA and **F** for CGGA).

To discover the relationship between GASCs and other stromal cells, we computed the levels of 14 stromal cells using the xCELL algorithm. Bar charts showed that endothelial cells, lymphatic endothelial cells, and microvascular endothelial cells were higher in the high-GASC group in all glioma population from the TCGA (**Figure 6A**) and CGGA (**Figure 6D**) databases. Univariate Cox regression revealed that the level of mesenchymal stem cells is a protective factor for glioma (**Figures 6B, E**). A coefficient matrix showed that the GASC score was positively correlated with the levels of endothelial cells, lymphatic endothelial cells, and microvascular endothelial cells (**Figures 6C, F**). Similar results were found in the HGG population (**Figure S3**).

### Immune Landscape of the High- and Low-GASC Groups

**Figure 7:**
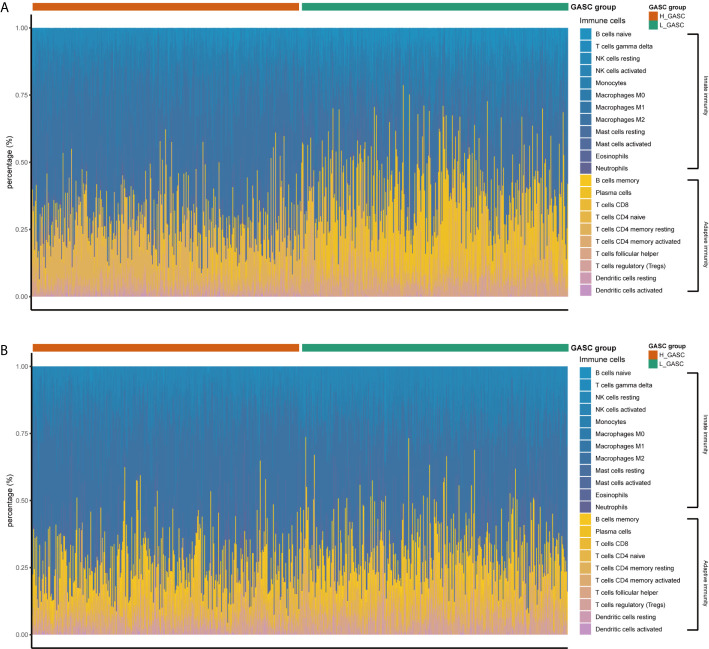
The relative abundances of the 22 types of immune cells. **(A)** Results from TCAG data. **(B)** Results from CGGA data.

**Figure 8:**
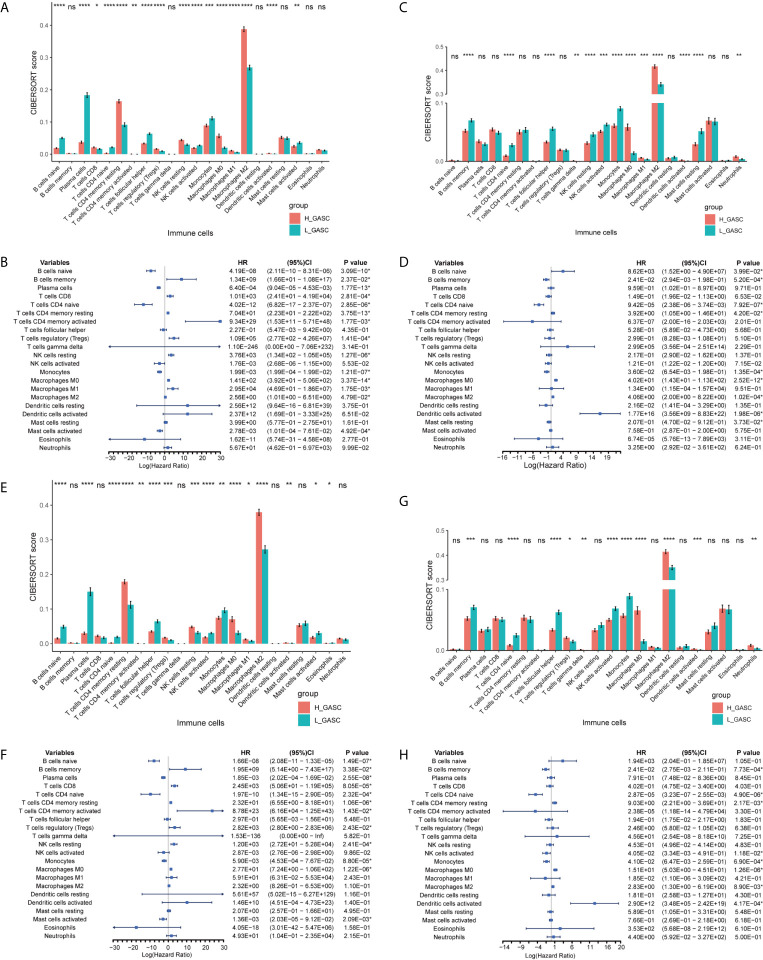
Associations between GASCs and immune cells. **(A, C, E, G)** Bar charts illustrating the differences in CIBERSORT scores between high- and low-GASC groups in all glioma population (**A** for TCGA and **C** for CGGA) and high-grade glioma population (**E** for TCGA and **G** for CGGA). **(B, D, F, H)** Forest plots of univariate Cox regression analysis of immune cells in all glioma population (**B** for TCGA and **D** for CGGA) and high-grade glioma population (**F** for TCGA and **H** for CGGA). ns: *p* ≥ 0.05, *p ≤ 0.05, **p ≤ 0.01, ***p ≤ 0.001, ****p ≤ 0.0001.

**Figure 9:**
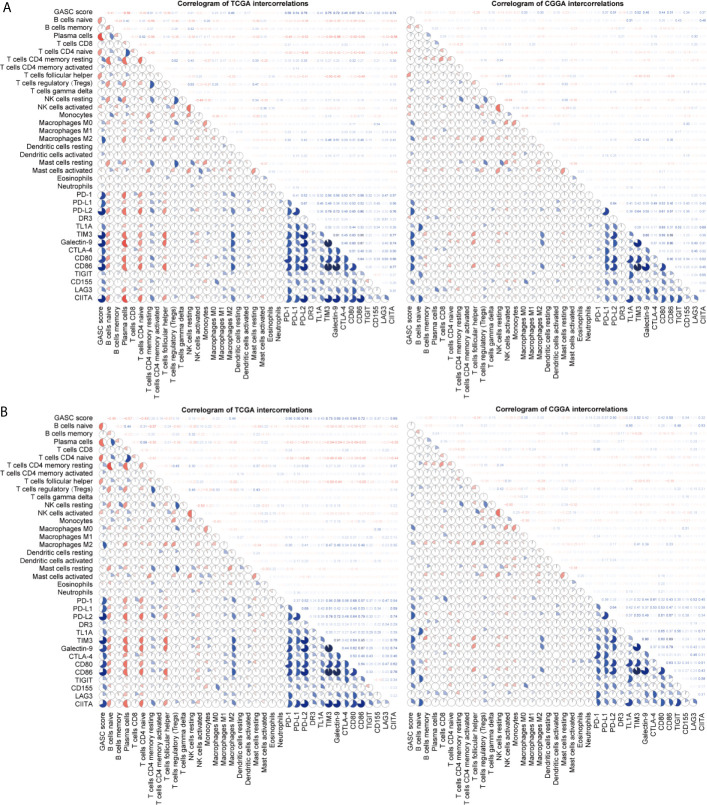
Correlogram of GASC score, immune cells, and expression of ICP intercorrelation. **(A)** Correlogram of data in all glioma population. **(B)** Correlogram of data in high-grade glioma population.

Because some immune-related terms were enriched in the functional annotation analysis, we explored the relationship between GASCs and the immune microenvironment. The CIBERSORT algorithm computed the relative abundance of 22 types of immune cells, which are shown in **Figure 7**. Overall, the adaptive immunity was at a relatively lower level in the high-GASC group compared with that in the low-GASC group. Notably, the level of M2 macrophages was significantly higher in the high-GASC group (**Figures 8A, C**) and was positively correlated with GASC score (**Figure 9A**) in all glioma population. Univariate Cox regression also revealed that the level of M2 macrophages is a risk factor for glioma (**Figures 8B, D**). Similar results were found in the HGG population (**Figures 8E–H**, **9B**).

**Figure 10:**
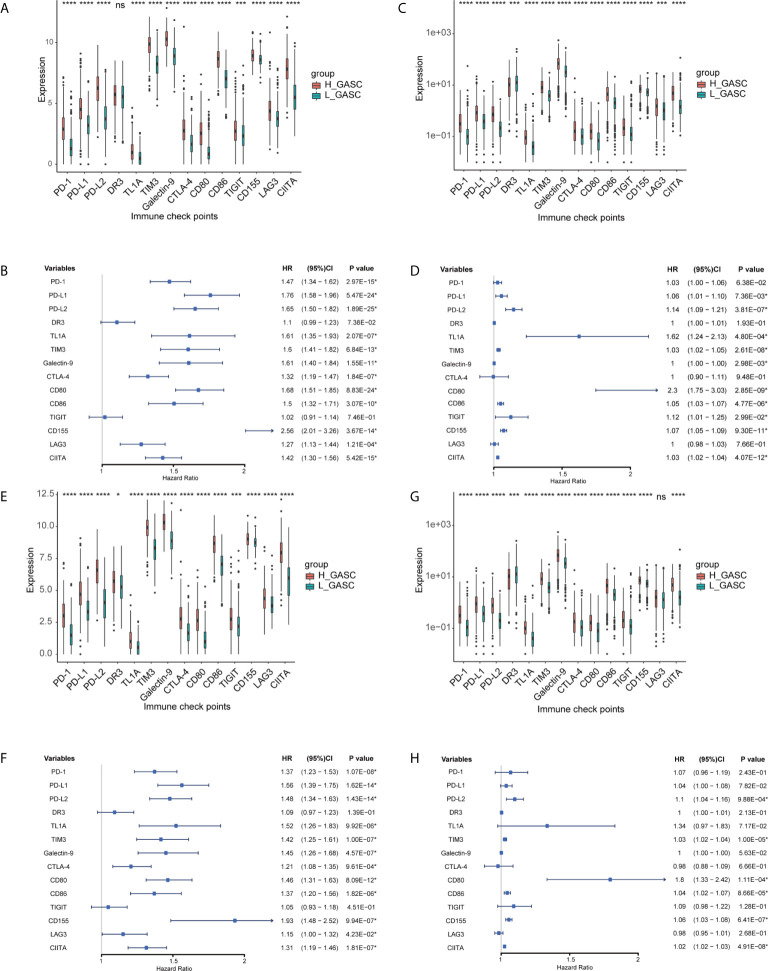
Associations between GASCs and expression of ICPs. **(A, C, E, G)** Bar charts illustrating the differences of ICP’ expressions between high- and low-GASC groups in all glioma population (**A** for TCGA and **C** for CGGA) and high-grade glioma population (**E** for TCGA and **G** for CGGA). **(B, D, F, H)** Forest plots of univariate Cox regression analysis of ICP’ expressions in all glioma population (**B** for TCGA and **D** for CGGA) and high-grade glioma population (**F** for TCGA and **H** for CGGA). ns: p > 0.05, *p ≤ 0.05, ***p ≤ 0.001, ****p ≤ 0.0001.

We also analyzed the correlation of GASCs and 14 important ICPs. As shown in **Figure 10**, the expression levels of most ICPs were statistically higher in the high-GASC group. The univariate Cox regression showed that the expression levels of PD-L2, TIM3, CD80, CD86, CD155, and CIITA were risk factors for glioma in all glioma population and the HGG population. Correlation analysis indicated strong positive correlations within ICPs. The GASC score was positively correlated with PD-1, PD-L1, PD-L2, TL1A, TIM3, Galactin-9, CTLA-4, CD80, CD86, CD155, LAG3, and CIITA in all glioma population and the HGG population (**Figure 9**).

### Copy Number Variations (CNVs) in DR3 and CIITA Indicated Worse OS

We also downloaded the somatic mutation and CNV data for glioma to analyze the difference in genomic alterations between the high- and low-GASC groups in all glioma population. The 20 genes with the greatest amounts of somatic mutations and CNVs are shown in **Figure S4**. We also compared the somatic mutations and CNVs of GASC markers between the high- and low-GASC groups (**Figure S5**), but found no significant difference in genomic alterations. However, in the comparison of somatic mutations and CNVs of ICPs between the high- and low-GASC groups (**Figure S6**), the results showed that the CNVs of DR3 and CIITA were significantly higher in the high-GASC group (**Figure S6E**). Survival analysis indicated that the CNVs of DR3 and CIITA significantly decreased the OS of glioma patients (**Figures S6F, G**).

### Higher THY1 and CD80 Methylation Indicated Better OS

In the search for a possible treatment target for glioma, we conducted methylation analysis of GASC markers and ICPs. Because there was not a satisfactory match between samples with methylation data in the CGGA database and samples in the CGGA_693 mRNA dataset, we also downloaded the CGGA_325 mRNA dataset and separated these samples into high- and low-GASC groups with the previously mentioned method. Overall, 26 glioma samples (6 in the high-GASC group and 20 in the low-GASC group) and 8 normal samples with methylation data were included.

**Figure 11:**
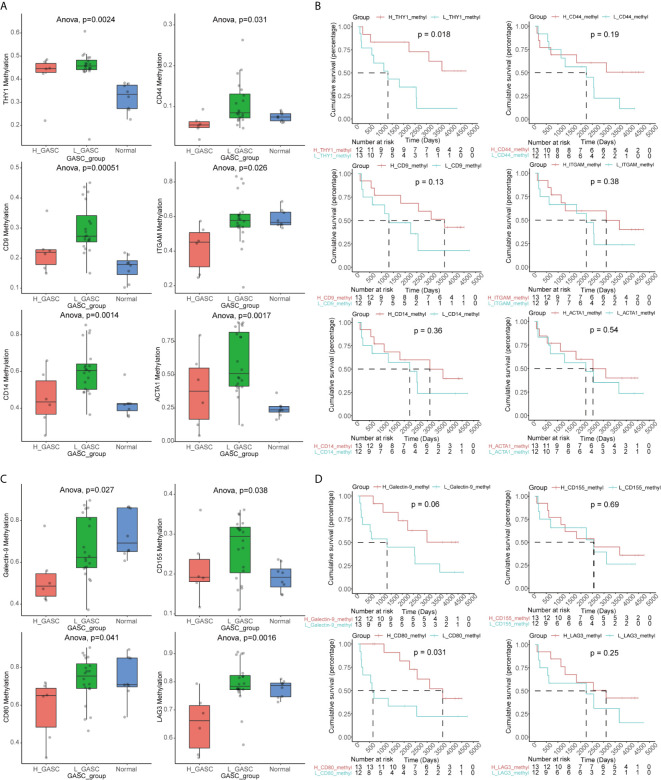
Methylation analysis of GASC markers and ICPs. **(A)** Box plots illustrating the differences in THY1, CD9, CD14, CD44, ITGAM and ACTA1 methylation levels across high-GASC, low-GASC and normal groups. **(B)** Kaplan-Meier overall survival (OS) curves for samples in high- and low-methylation groups of THY1, CD9, CD14, CD44, ITGAM and ACTA1. **(C)** Box plots illustrating the differences in Galectin-9, CD80, CD155 and LAG3 methylation levels across high-GASC, low-GASC and normal groups. **(D)** Kaplan-Meier OS curves for samples in high- and low-methylation groups of Galectin-9, CD80, CD155 and LAG3.

The GASC markers indicated that the methylation levels of THY1, CD9, CD14, CD44, ITGAM, and ACTA1 were significantly different among the high-GASC, low-GASC, and normal groups (**Figure 11A** and **Figure S7**). Then, we divided the glioma samples into high- and low-methylation groups according to the median of the gene methylation level. Survival analysis indicated that statistical difference was only observed between high- and low-THY1 methylation groups (*p* = 0.018; **Figure 11B**). High THY1 methylation suggested greater patient OS. In the methylation analysis of ICPs, significant differences were detected in Galactin-9, CD80, CD155, and LAG3 (**Figure 11C** and **Figure S8**). However, only high- and low-CD80 methylation groups showed statistical difference in the survival analysis (*p* = 0.031; **Figure 11D**), and high CD80 methylation indicated better OS.

### Predicted Potential Immunotherapy Responses Between the High- and Low-GASC Groups

The TIDE webtool was applied to predict the likelihood of immune response for each sample. The results showed that in all glioma population, the low-GASC group (56%, 197/351 in TCGA; 40%, 138/347 in CGGA) was more likely to respond to immunotherapy than the high-GASC group (40%, 141/351 in TCGA; 29%, 102/346 in CGGA). However, in the HGG population, difference was found only in the TCGA dataset (high-GASC vs. low-GASC: 31% vs. 44%; **Figures S9A–E**).

In order to further analyze the immune infiltration between the responder and no responder groups, we compared the levels of immune cells between these two groups. The results showed that in all glioma population, the responder group had lower “T cells CD8” and “Macrophages M0” and higher “Mast cells activated” (**Figures S9F, G**). For the HGG population, “T cells CD8” was lower in the responder group, and “Mast cells activated” was higher in the responder group (**Figures S9H, I**).

### Construction of a Risk Score System and Establishment and Validation of a Nomogram Survival Model

**Figure 12:**
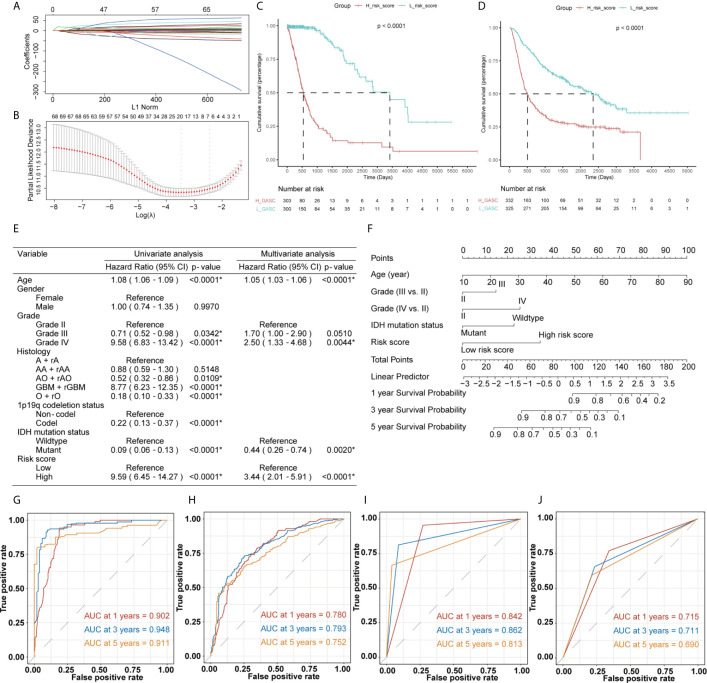
Construction of the risk score system and establishment and validation of the nomogram survival model. **(A, B)** LASSO Cox regression analysis of training dataset. **(C, D)** Kaplan-Meier overall survival (OS) curves for samples in high- and low-risk score groups (**C** for training dataset and **D** for validation dataset). **(E)** Uni- and multi-variate Cox regression analysis for prognostic model. *Statistical significance. **(F)** The nomogram for predicting 1-, 3‐, or 5‐year OS. **(G, H)** Time-dependent ROC curves of the nomogram prediction on the 1-, 3-, and 5-year survival rates (**G** for training dataset and **H** for validation dataset). **(I, J)** Time-dependent ROC curves of the IDH mutation status prediction on the 1-, 3-, and 5-year survival rates (**I** for training dataset and **J** for validation dataset).

The mRNA sequencing data from TCGA (702 samples) was used as training dataset, and the data from CGGA (693 samples) was set as an independent validation dataset. For the training dataset, GASC score, GASC markers, immune checkpoints, stemness score, mesenchymal-EMT score, tumorigenic cytokine score, angiogenic activity score, stromal cell scores, and immune cell scores were filtered using LASSO regression with the “glmnet” R package. The change in trajectory of each variable was plotted in **Figure 12A**. We utilized 10-fold cross-validation to construct the model, and **Figure 12B** shows the confidence interval under each lambda. When lambda equaled 0.03431609, the model reached the optimal value, and 19 variables were selected for the next analysis. In the multivariable Cox regression analysis, the number of variables was reduced to 9, and the final 9-variable signature formula was: Risk score = 0.20 × CSPG4 – 0.30 × ALCAM + 35.16 × Adipocytes – 11.11 × Osteoblast – 7.05 × Pericytes – 2.50 × Plasma cells + 0.22 × CD274 + 0.16 × CD80 + 13.45 × angiogenesis. The risk score was calculated for each sample in the training and validation datasets. Thus, we divided samples into high- and low-risk groups according to the median risk score. Survival analysis revealed that in the training dataset, glioma patients in high-risk group have worse OS (*p* < 0.0001; **Figure 12C**), which was also confirmed in the validation dataset (*p* < 0.0001; **Figure 12D**).

Then, we constructed a nomogram model to predict the prognosis of glioma, which included the risk score and clinicopathologic features. The uni- and multivariate Cox regression analysis (**Figure 12E**) indicated that risk score was an independent predictor for glioma prognostics. We finally included four features (age, WHO grade, isocitrate dehydrogenase (IDH) mutation status, and risk score) in the nomogram model (**Figure 12F**). Time-dependent ROC analysis further indicated that the area under the curve (AUC) for 1-, 3-, and 5-year OS were 0.902, 0.948, and 0.911, respectively, in the training dataset (**Figure 12G**). These AUCs were better compared with IDH mutation status, which is a traditional indicator, and were 0.842, 0.862, and 0.813 at the 1-, 3-, and 5-year marks (**Figure 12I**). Similar results were obtained in the validation datasets (**Figures 12H, J**).

## Discussion

The GASC is a recently identified particular type of cell in the glioma microenvironment, with various names, e.g., glioma-associated human MSCs (GA-hMSCs) (7). The phenotypic and functional properties of GASCs are similar to those of cancer-associated fibroblasts and mesenchymal stem cells. The mechanism of GASCs in the glioma microenvironment is still largely unknown. In this work, we explored the potential mechanisms of GASCs in the glioma microenvironment, and discovered that GASCs may upregulate the level of M2 macrophages and ICPs. We also found that the CNVs of DR3 and CIITA were higher in the high-GASC group, and the methylation level of THY1 was lower in the high-GASC group, which could be a potential treatment target for glioma, particularly in HGG.

The tumor microenvironment determines the invasiveness of glioma. The EMT regulates this invasive state of glioma, particularly in HGG (9). Studies reported that GASCs drive cell invasion through HA synthase-2 (HAS2) induction (15), the UCA1/miR-182/PFKFB2 axis (16), the C5a/p38/ZEB1 axis (17) and CCL2/JAK1/MLC2 signaling (18). In the current work, we also discovered that the GASC score was positively correlated with the mesenchymal-EMT score in all glioma population and the HGG population (**Figure 5**). Terms from functional annotation include adhesion-related terms (**Tables S2**, **S6**), e.g., “Cell adhesion molecules”.

In the current study, we found a strong correlation between the GASC score and tumorigenic cytokine score, indicating the tumor-supporting function of GASCs. Studies reported that GASCs have tumor-promoting effects *in vitro* and *in vivo (* 19–21). Additionally, Figueroa et al. also suggested that the tumor-supporting role of GASCs is mediated by the exosomal delivery of specific oncogenic miRNAs (21).

Although GASCs infiltrate into the glioma stroma, they are predominantly located around blood vessels (22), particularly abnormal vessels (23). Previous studies indicated that GASCs increase the angiogenesis of glioma (24, 25). Zhang et al. suggested that CD90^low^ (THY1) GASCs stimulate angiogenesis *via* vascular endothelial cells (25). In the current work, we detected a high correlation between the GASC score and the angiogenesis score (**Figure 5**) in all glioma population and the HGG population. We also found that in addition to endothelial cells, the GASC score also was positively correlated with microvascular endothelial cells (**Figures 6** and **Figure S3**). The levels of endothelial cells and microvascular endothelial cells were higher in the high-GASC group (**Figures 6** and **Figure S3**). These results indicated that GASCs may promote angiogenesis of glioma by stimulating the growth of both blood vessels and microvessels, which requires further verification.

Tumor-associated macrophages (TAMs) play an emerging role in glioma progression and are found in high proportions in the immune landscape of malignant glioma (26, 27). There are continuous phenotypes in the activation state of TAMs, in which M1 and M2 represent two extreme phenotypes (28). M2 has an anti-inflammatory phenotype, which leads to downregulation of immune responses, and thus prevents tissue damage and supports healing processes (27). In this work, our results suggest for the first time that GASCs are highly correlated with M2 macrophages in the glioma microenvironment. Based on our results, the level of M2 macrophages in the high-GASC group is statistically higher than that in the low-GASC group in all glioma population and the HGG population (*p* ≤ 0.0001; **Figure 8**). We also found high correlation coefficients between GASC scores and M2 macrophages in all glioma population (R = 0.46 (TCGA); R = 0.30 (CGGA); **Figure 9A**) and the HGG population (R = 0.44 (TCGA); R = 0.26 (CGGA); **Figure 9B**). These results indicated that TAMs may be phenotypically polarized to M2 macrophages by GASCs, which may further depress the immunity of the microenvironment and stimulate malignant progression of glioma. Conversely, the M2 macrophages may also upregulate the level of GASCs and further increase the malignant properties of glioma, e.g., invasion and angiogenesis.

Immune checkpoint blockade is the most developed immunotherapy in clinical use (4), but its efficiency still remains doubtful. We analyzed the expression levels of 14 important ICPs and found that the expression levels of most ICPs were higher in the high-GASC group. Although this result suggested that the high-GASC group may have a more optimal immunotherapy response, the results from the TIDE prediction were puzzling because they showed a contrary tendency (**Figure S9**). These contradictory results reflect the inner complexity of glioma, and in response to this, further high-quality studies of immunotherapy in glioma are required.

McDonald et al. reported that deletion of DR3 (Tumor Necrosis Factor Receptor Superfamily Members 25, TNFRSF25) was found in oligodendroglioma (29). The results from Qian et al.’s work suggested that suppression of CIITA (class II transactivator) downregulates the expression of MHC class II molecules in glioma (30). In the current study, we discovered that the CNVs of DR3 (P < 0.001) and CIITA (*p* = 0.015) were significantly higher in the high-GASC group (**Figure S6E**). The glioma patients with amplified/deleted DR3 or amplified CIITA had worse OS compared with wild-type glioma patients (**Figures S6F, G**). These results indicate that the CNVs of DR3 and CIITA may be potential prognostic indicators for glioma, and further studies are expected to verify their efficiency.

THY1 (CD90) is a surrogate marker for a variety of stem cells, including glioblastoma stem cells (GSC) (31) and GASC (7). Svensson et al. detected CD90^-^ and CD90^+^ GASC subpopulations by cell sorting and discovered that the CD90^-^ subpopulation exhibited greater tumor vascularization and immunosuppression activity than the CD90^+^ subpopulation (32). Zhang et al. further investigated these two subpopulations. They found that CD90^high^ GASCs drove glioma progression *via* increasing proliferation, migration, and adhesion. However, CD90^low^ GASCs contributed to glioma progression through the stimulation of vascular formation *via* vascular endothelial cells (25). In the current work, we discovered that the methylation levels were different among high-GASC, low-GASC, and normal groups (**Figure 11A**), and the high THY1 methylation group had better OS compared with the low THY1 methylation group (*p* = 0.018; **Figure 11B**). These results suggested for the first time that the methylation of THY1 could be a potential prognostic indicator of glioma as well as a treatment target.

To create a comprehensive risk score, we included the following features, produced from mRNA sequencing data, in the filtering process: GASC score, stemness score, mesenchymal-EMT score, tumorigenic cytokine score, angiogenic activity score, stromal cell scores, and immune cell scores. The results showed that the risk score could be used to differentiate patients with high or low risk (**Figures 12C, D**), and the risk score was an independent prognostic indicator for glioma (**Figure 12E**). The validation results from the CGGA validation dataset verified the robustness of our nomogram model (**Figure 12H**). We also compared the efficiency of the nomogram model with a traditional prognostic indicator, IDH mutation status. The AUCs for the nomogram model were better than those for IDH mutation status in the training dataset (**Figures 12G, I**) and the validation dataset (**Figures 12H, J**). To verify the credibility of this nomogram model, further high-quality clinical studies are required.

There are limitations in our study. First, because of a lack of public mRNA resources with proportions of GASCs, stromal cells, and immune cells, we selected the ssGEAS algorithm to compute these data, because it has been widely used with proven reliability. Second, because this is a retrospective study, the efficiency of our risk score and nomogram model needs to be verified in further high-quality prospective cohorts. In addition, our predicted results for immunotherapy response were contradictory with the expression level of ICPs. Glioma, particularly HGG, is characterized by remarkably high tumor heterogeneity and an “immune-cold” phenotype, denoting an immunosuppressive microenvironment. The GASC is an important cell type in the microenvironment and might influence immunotherapy responses. Based on previous research, the overexpression of some ICPs on malignant cells may increase the anti-tumor immune responses (33). Nevertheless, although our data suggested that higher ICP expression occurs in the high-GASC group, TIME prediction revealed worse immunotherapy responses in the high-GASC group. This contradiction may result from the inner complexity of the glioma microenvironment. Further prospective clinical trials to test immunotherapy for glioma with the GASC proportion data are required to produce a reliable conclusion regarding the relationship between GASCs and immunotherapy responses.

## Conclusion

We found potential mechanisms of GASCs in the glioma microenvironment, particularly HGG, and we also discovered that GASCs are positively correlated with the level of M2 macrophages and ICPs. The methylation of THY1 decreased in the high-GASC group, which could be a prognostic indicator and treatment target for glioma. We also developed a prognostic nomogram for glioma. Further studies to verify these findings and the performance of our model with large prospective cohorts are warranted.

## Data Availability Statement

Publicly available datasets were analyzed in this study. This data can be found here: TCGA database (https://portal.gdc.cancer.gov/) and CGGA database (www.cgga.org.cn/).

## Author Contributions

CM conceived and designed the investigation. XC and FY analyzed the data and drafted the manuscript. JZ, JY, CT, and ZC conducted statistical analyses. All authors contributed to the article and approved the submitted version.

## Conflict of Interest

The authors declare that the research was conducted in the absence of any commercial or financial relationships that could be construed as a potential conflict of interest.

## Supplementary Material

Supplementary Figure 1 Differential analysis of expressed genes. **(A, B)** Heatmap of DEGs in all glioma population (**A** for TCGA and **B** for CGGA). **(C, D)** Heatmap of DEGs in high-grade glioma population (**C** for TCGA and **D** for CGGA).

Supplementary Figure 2 Functional annotation of downregulated DEGs between the high- and low-GASC groups. **(A, B)** GO analysis **(A)** and KEGG pathway analysis (**B**) of down-regulated DEGs in all glioma population from TCGA data. **(C, D)** GO analysis **(C)** and KEGG pathway analysis **(D)** of down-regulated DEGs in all glioma population from CGGA data. **(E, F)** GO analysis (**E**) and KEGG pathway analysis **(F)** of down-regulated DEGs in high-grade glioma population from TCGA data. **(G, H)** GO analysis **(G)** and KEGG pathway analysis **(H)** of down-regulated DEGs in high-grade glioma population from CGGA data.

Supplementary Figure 3 Associations between GASCs and stromal cells in the high-grade glioma population. **(A, D)** Bar chart illustrating the differences in xCELL scores between high- and low-GASC groups (**A** for TCGA and **D** for CGGA). ns: *p* > 0.05, **p* ≤ 0.05, ***p* ≤ 0.01, ****p* ≤ 0.001, *****p* ≤ 0.0001. **(B,E)** Forest plot of univariate Cox regression analysis of stromal cells (**B** for TCGA and **E** for CGGA). ** (C,F)** Correlogram of GASC score and stromal cells intercorrelation (**C** for TCGA and **F** for CGGA).

Supplementary Figure 4 Comparison of genomic alterations between the high- and low-GASC groups in the TCGA dataset. **(A, B)** Differential copy number variation analysis between high- **(A)** and low- **(B)** GASC groups. **(C, D)** Differential somatic mutation analysis between high- **(C)** and low- **(D)** GASC groups.

Supplementary Figure 5 Comparison of genomic alterations of GASC markers between the high- and low-GASC groups in the TCGA dataset. **(A, B)** Differential copy number variation analysis between high- **(A)** and low- **(B)** GASC groups. **(C, D)** Differential somatic mutation analysis between high- **(C)** and low- **(D)** GASC groups. **(E)** Correlations between GASC groups and somatic mutation of GASC markers.

Supplementary Figure 6 Comparison of genomic alterations of ICPs between the high- and low-GASC groups in the TCGA dataset. **(A, B)** Differential copy number variation analysis between high- **(A)** and low- (**B**) GASC groups. **(C, D)** Differential somatic mutation analysis between high- **(C)** and low- (**D**) GASC groups. **(E)** Correlations between GASC groups and somatic mutation of ICPs. **(F,G)** Kaplan-Meier overall survival (OS) curves for samples of amplified, deleted and wildtype DR3 groups **(F)** and CIITA groups **(G)**.

Supplementary Figure 7 Methylation analysis of GASC markers. **(A)** Box plots illustrating the differences in CD34, ALCAM, CSPG4, ENG, GFAP, S100A4, NT5E, PDGFRB, PECAM1 and PTPRC methylation levels across high-GASC, low-GASC and normal groups.

Supplementary Figure 8 Methylation analysis of immune checkpoints. **(A)** Box plots illustrating the differences in PD-1, PD-L1, PD-L2, DR3, TL1A, CTLA-4, CD86, TIM3 and CIITA methylation levels across high-GASC, low-GASC and normal groups.

Supplementary Figure 9 Predicted potential immunotherapy responses between the high- and low-GASC groups. **(A–D)** Predicted potential immunotherapy responses of samples from all glioma population (**A** for TCGA and **B** for CGGA) and high-grade glioma population (**C** for TCGA and **D** for CGGA). **(E)** Correlation of GASC and Predicted immunotherapy responses. **(F–I)** Bar chart illustrating the differences in immune cell scores between responder and no responder groups in all glioma population (**F** for TCGA and **G** for CGGA) and high-grade glioma population (**H** for TCGA and **I** for CGGA).
