# Supplementary figures and images for: Glioma-Associated Stromal Cells Stimulate Glioma Malignancy by Regulating the Tumor Immune Microenvironment
Source: Front Oncol. 2021 Apr 29;11:672928. doi: 10.3389/fonc.2021.672928 (PMC8117153; doi:10.3389/fonc.2021.672928)

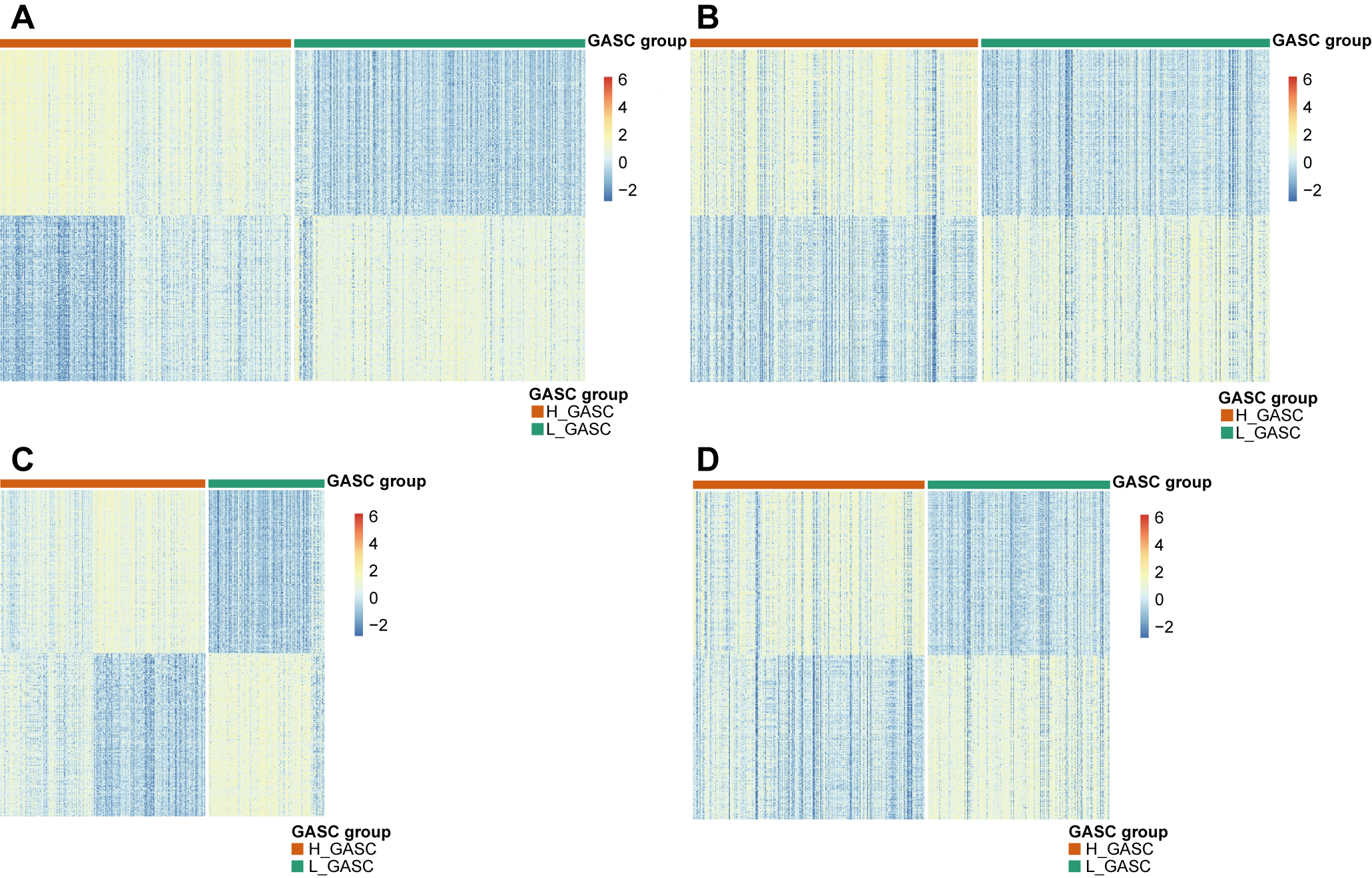

Supplement: Supplementary Figure 1 — Differential analysis of expressed genes. (A, B) Heatmap of DEGs in all glioma population (A for TCGA and B for CGGA). (C, D) Heatmap of DEGs in high-grade glioma population (C for TCGA and D for CGGA). [file Image_1.tif]

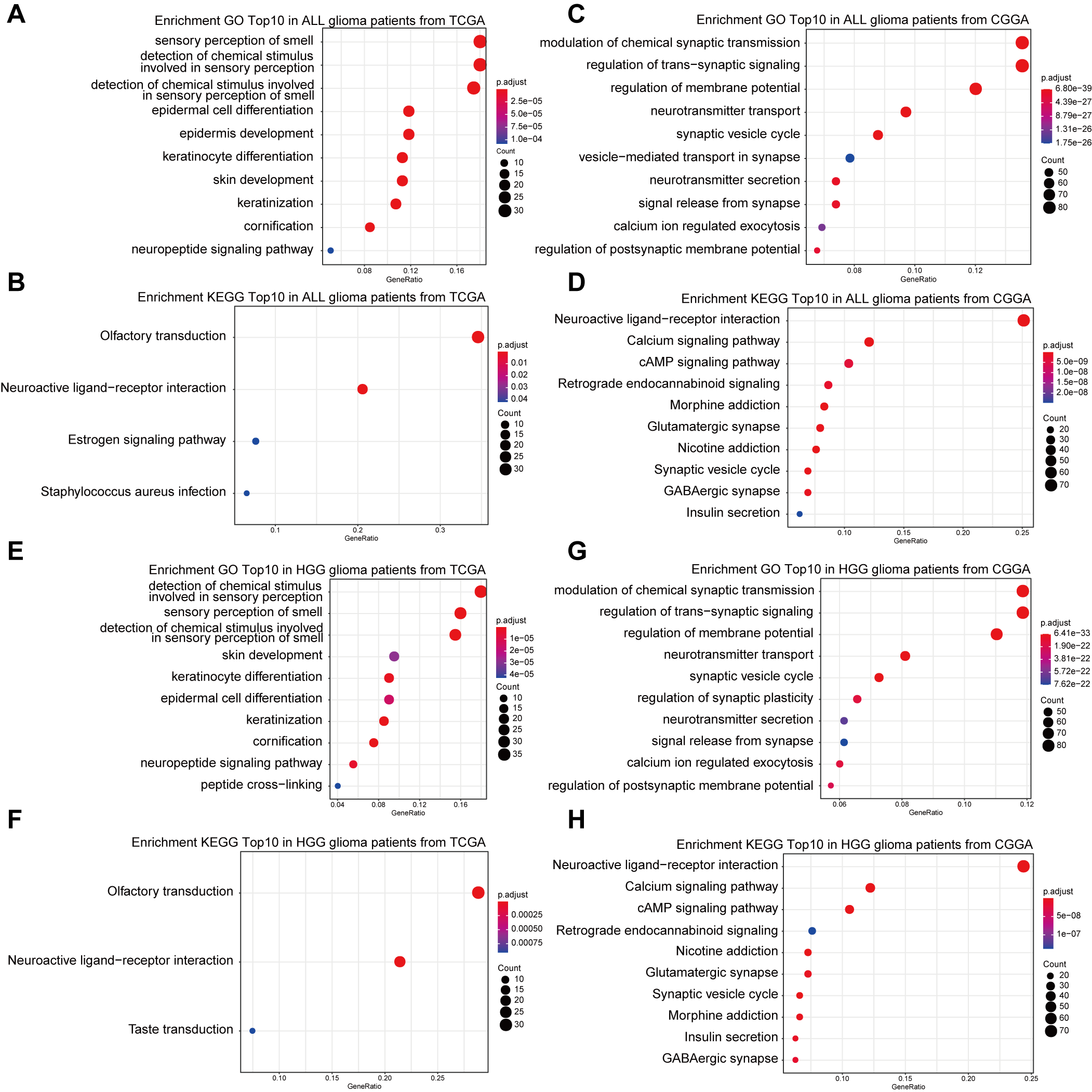

Supplement: Supplementary Figure 2 — Functional annotation of downregulated DEGs between the high- and low-GASC groups. (A, B) GO analysis (A) and KEGG pathway analysis (B) of down-regulated DEGs in all glioma population from TCGA data. (C, D) GO analysis (C) and KEGG pathway analysis (D) of down-regulated DEGs in all glioma population from CGGA data. (E, F) GO analysis (E) and KEGG pathway analysis (F) of down-regulated DEGs in high-grade glioma population from TCGA data. (G, H) GO analysis (G) and KEGG pathway analysis (H) of down-regulated DEGs in high-grade glioma population from CGGA data. [file Image_2.tif]

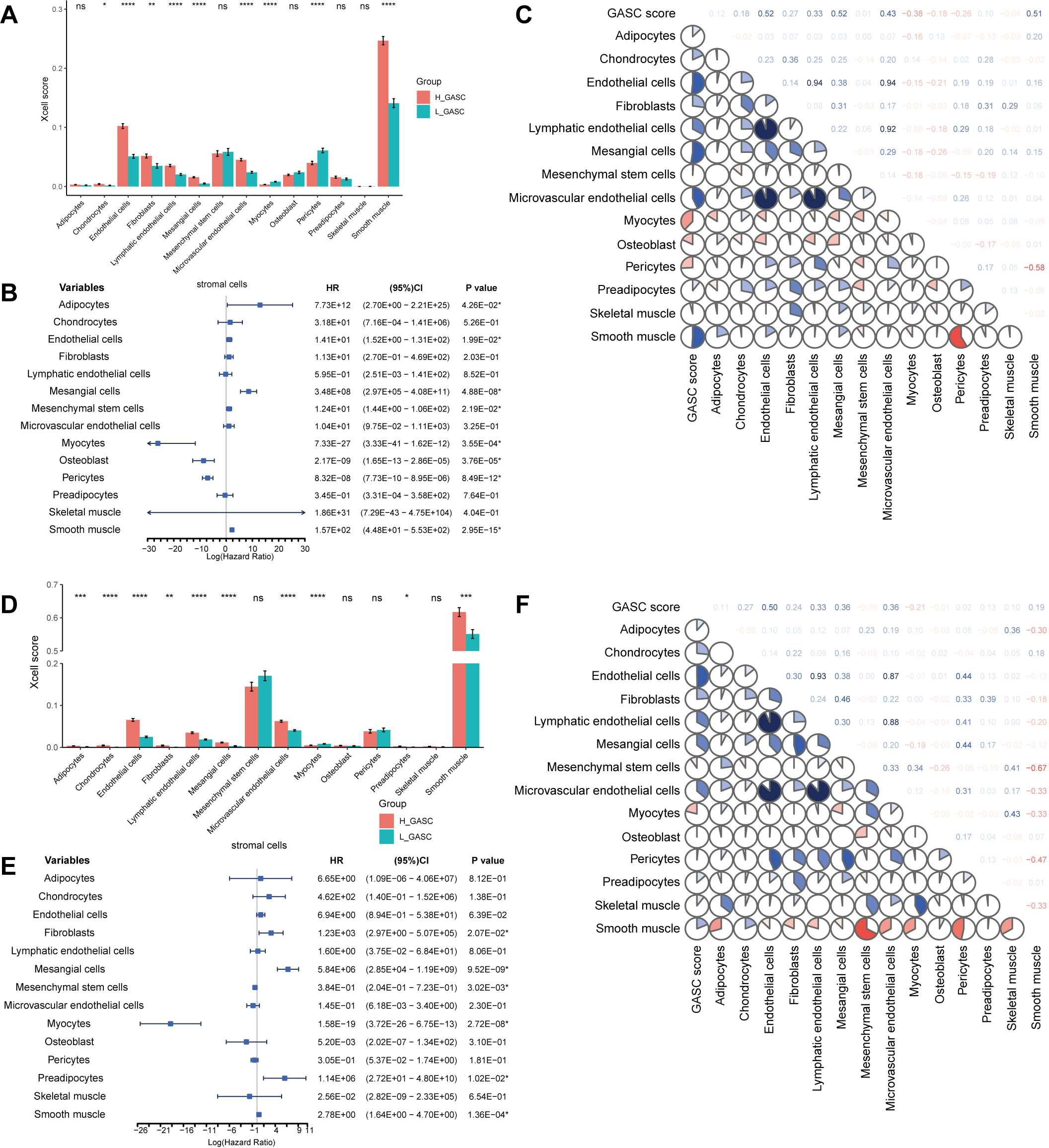

Supplement: Supplementary Figure 3 — Associations between GASCs and stromal cells in the high-grade glioma population. (A, D) Bar chart illustrating the differences in xCELL scores between high- and low-GASC groups (A for TCGA and D for CGGA). ns: p > 0.05, *p ≤ 0.05, **p ≤ 0.01, ***p ≤ 0.001, ****p ≤ 0.0001. (B,E) Forest plot of univariate Cox regression analysis of stromal cells (B for TCGA and E for CGGA). (C,F) Correlogram of GASC score and stromal cells intercorrelation (C for TCGA and F for CGGA). [file Image_3.tif]

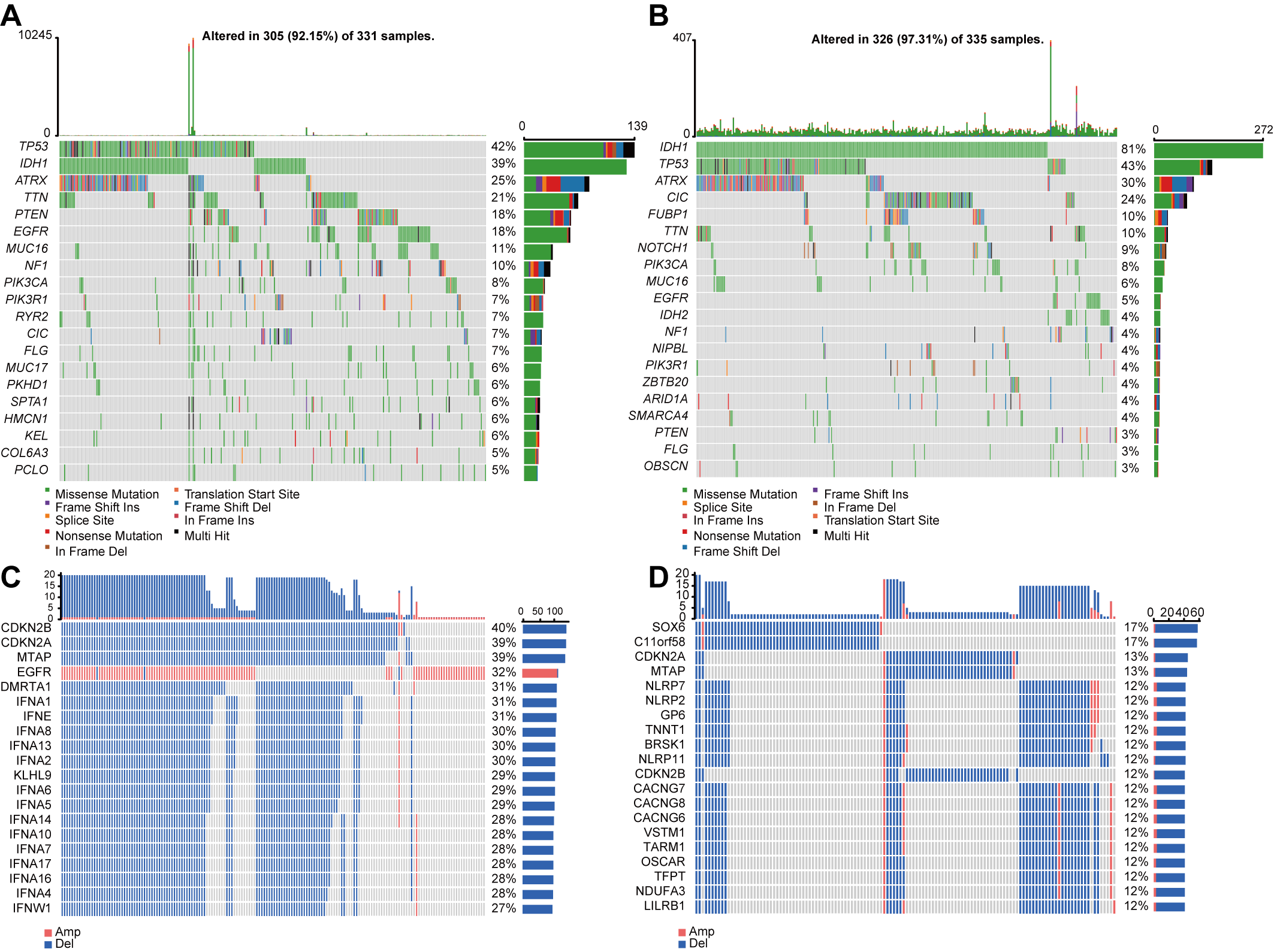

Supplement: Supplementary Figure 4 — Comparison of genomic alterations between the high- and low-GASC groups in the TCGA dataset. (A, B) Differential copy number variation analysis between high- (A) and low- (B) GASC groups. (C, D) Differential somatic mutation analysis between high- (C) and low- (D) GASC groups. [file Image_4.tif]

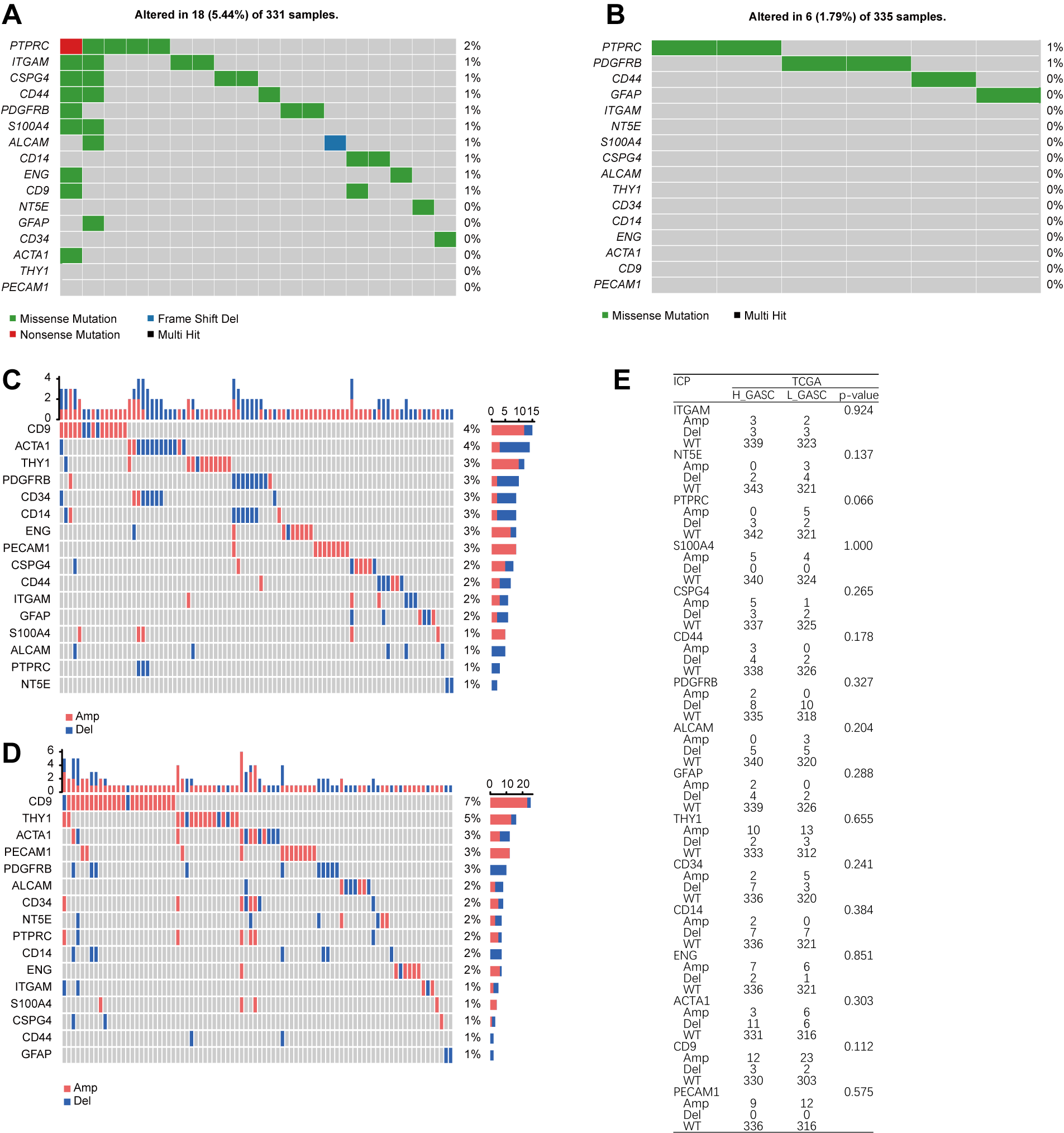

Supplement: Supplementary Figure 5 — Comparison of genomic alterations of GASC markers between the high- and low-GASC groups in the TCGA dataset. (A, B) Differential copy number variation analysis between high- (A) and low- (B) GASC groups. (C, D) Differential somatic mutation analysis between high- (C) and low- (D) GASC groups. (E) Correlations between GASC groups and somatic mutation of GASC markers. [file Image_5.tif]

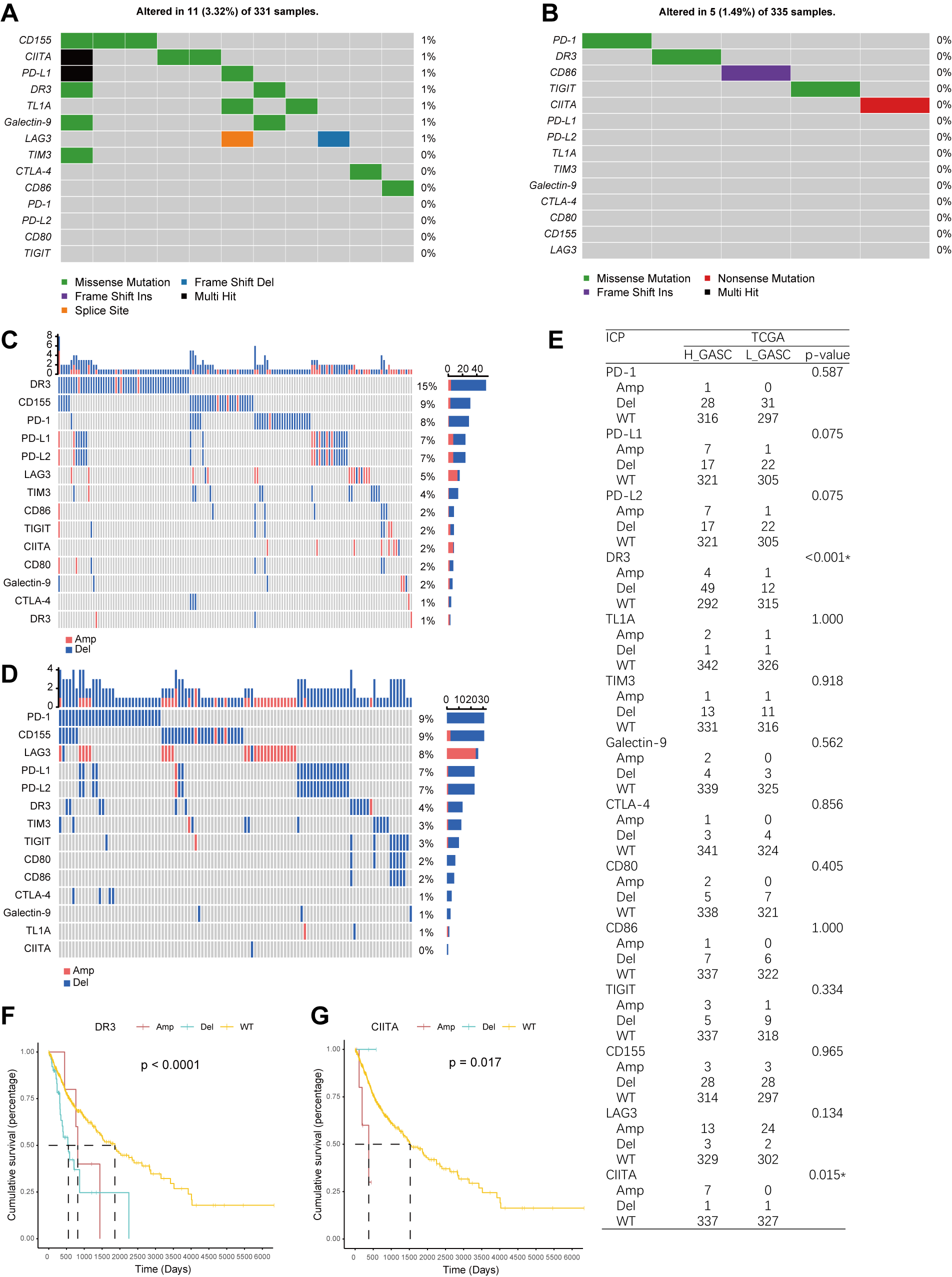

Supplement: Supplementary Figure 6 — Comparison of genomic alterations of ICPs between the high- and low-GASC groups in the TCGA dataset. (A, B) Differential copy number variation analysis between high- (A) and low- (B) GASC groups. (C, D) Differential somatic mutation analysis between high- (C) and low- (D) GASC groups. (E) Correlations between GASC groups and somatic mutation of ICPs. (F,G) Kaplan-Meier overall survival (OS) curves for samples of amplified, deleted and wildtype DR3 groups (F) and CIITA groups (G). [file Image_6.tif]

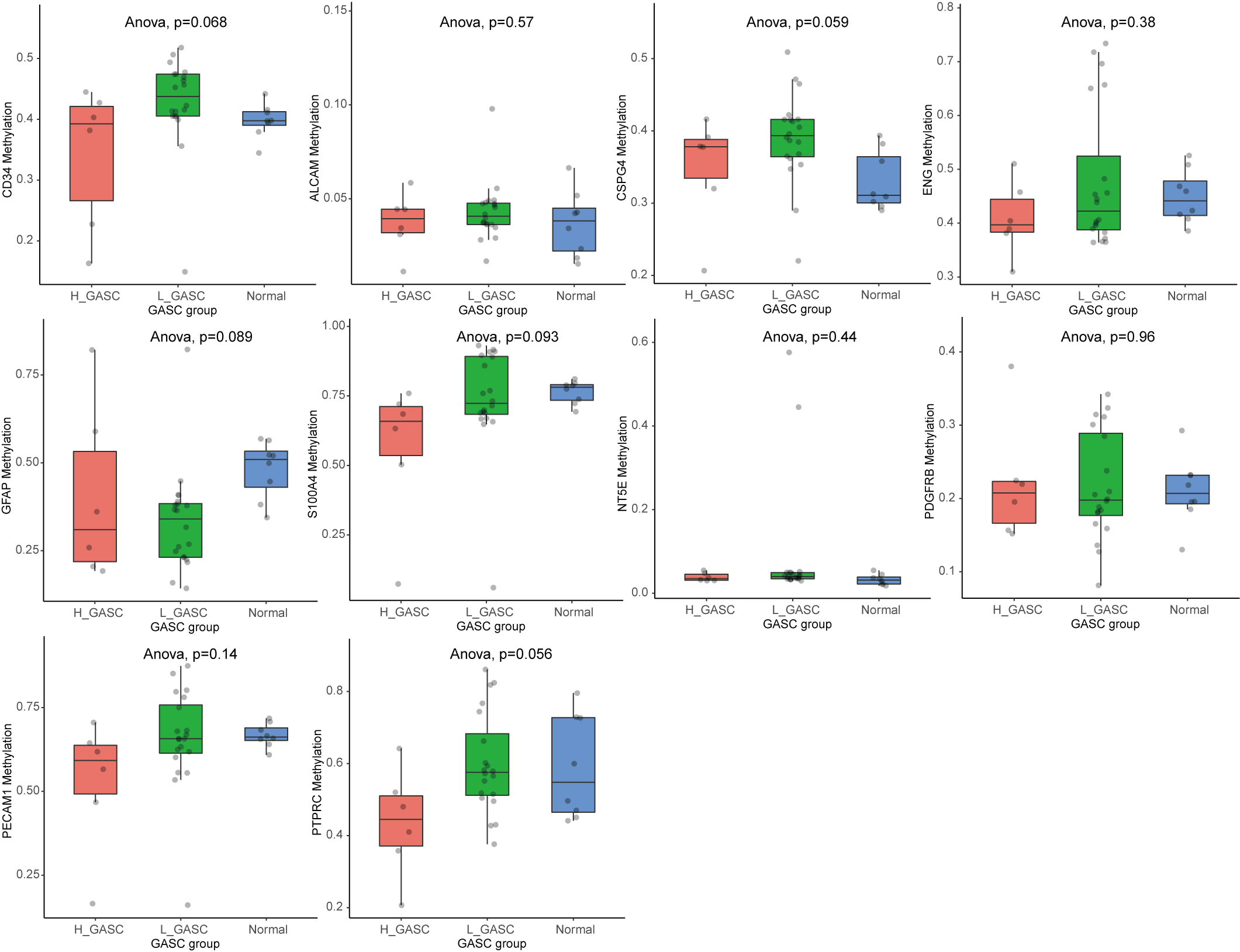

Supplement: Supplementary Figure 7 — Methylation analysis of GASC markers. (A) Box plots illustrating the differences in CD34, ALCAM, CSPG4, ENG, GFAP, S100A4, NT5E, PDGFRB, PECAM1 and PTPRC methylation levels across high-GASC, low-GASC and normal groups. [file Image_7.tif]

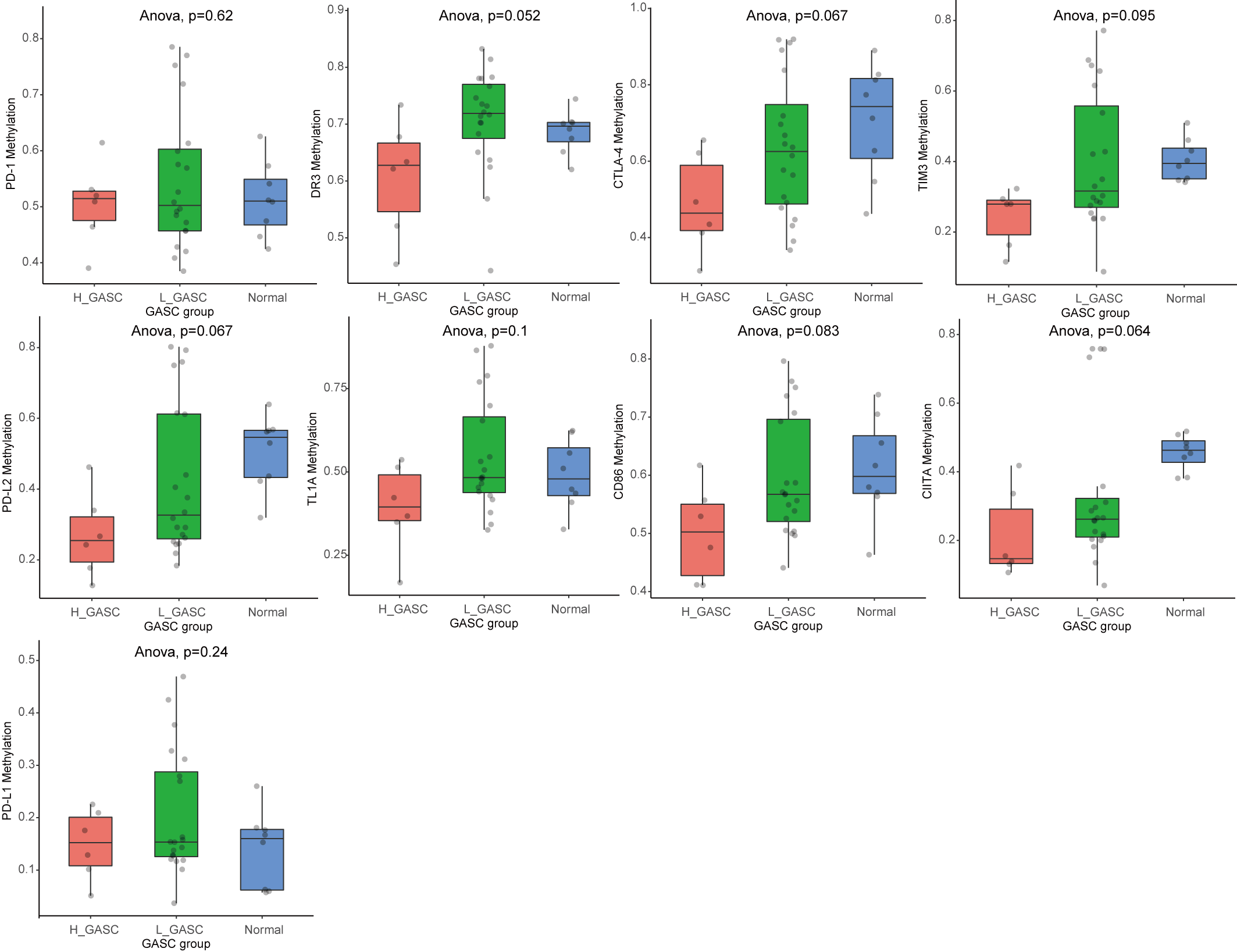

Supplement: Supplementary Figure 8 — Methylation analysis of immune checkpoints. (A) Box plots illustrating the differences in PD-1, PD-L1, PD-L2, DR3, TL1A, CTLA-4, CD86, TIM3 and CIITA methylation levels across high-GASC, low-GASC and normal groups. [file Image_8.tif]

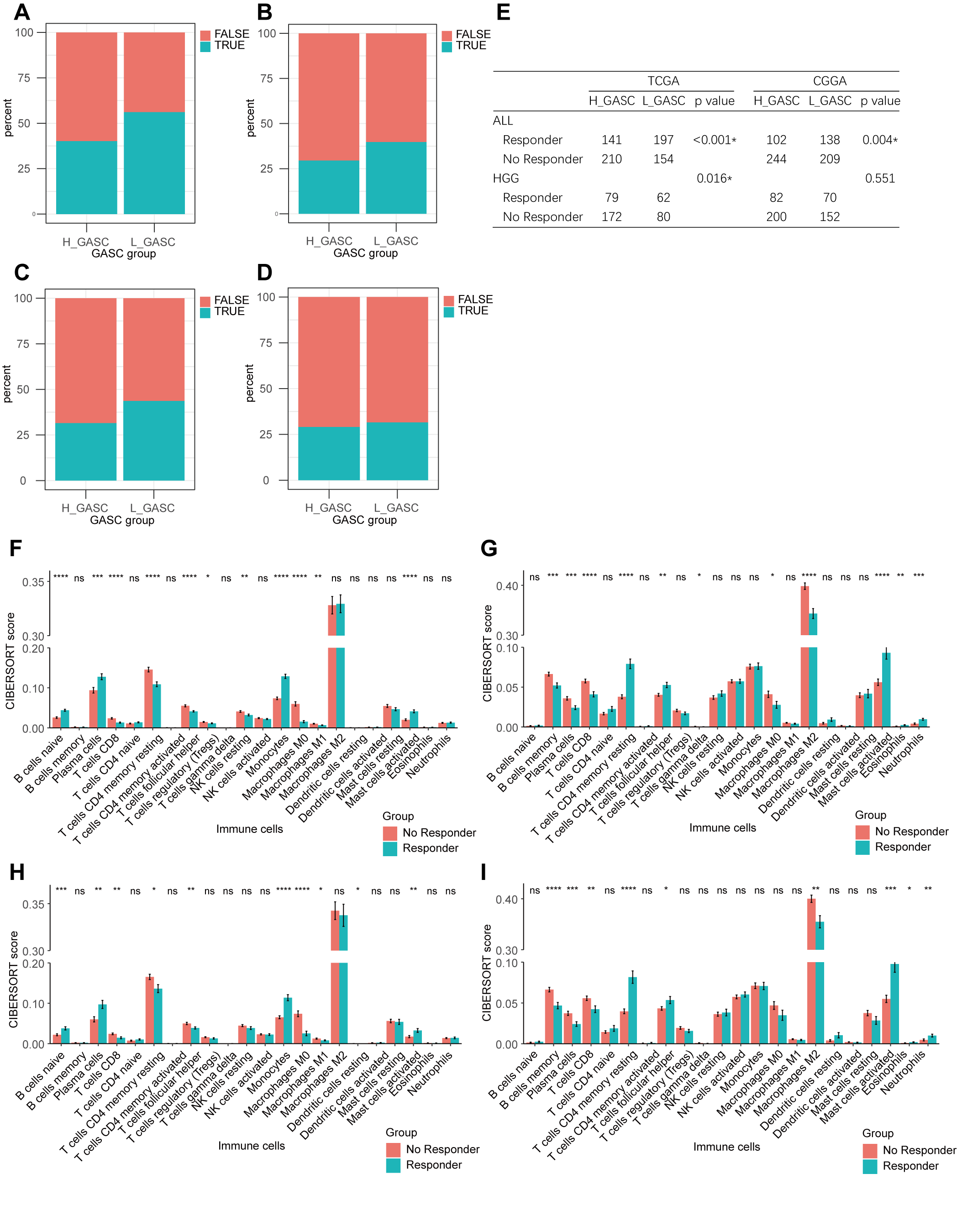

Supplement: Supplementary Figure 9 — Predicted potential immunotherapy responses between the high- and low-GASC groups. (A–D) Predicted potential immunotherapy responses of samples from all glioma population (A for TCGA and B for CGGA) and high-grade glioma population (C for TCGA and D for CGGA). (E) Correlation of GASC and Predicted immunotherapy responses. (F–I) Bar chart illustrating the differences in immune cell scores between responder and no responder groups in all glioma population (F for TCGA and G for CGGA) and high-grade glioma population (H for TCGA and I for CGGA). [file Image_9.tif]
